# The added value of genetic information in colorectal cancer risk prediction models: development and evaluation in the UK Biobank prospective cohort study

**DOI:** 10.1038/s41416-018-0282-8

**Published:** 2018-10-16

**Authors:** Todd Smith, Marc J. Gunter, Ioanna Tzoulaki, David C. Muller

**Affiliations:** 1 Department of Epidemiology and Biostatistics, School of Public Health, Imperial College London, London, W2 1PG UK; 2 MRC-PHE Centre for Environment and Health, School of Public Health, Imperial College London, London, W2 1PG UK; 3 Section of Nutrition and Metabolism, International Agency for Research on Cancer, Lyon, France; 4 Department of Hygiene and Epidemiology, University of Ioannina Medical School, Ioannina, Greece

## Abstract

Colorectal cancer (CRC) risk prediction models could be used to risk-stratify the population to provide individually tailored screening provision. Using participants from the UK Biobank prospective cohort study, we evaluated whether the addition of a genetic risk score (GRS) could improve the performance of two previously validated models. Inclusion of the GRS did not appreciably improve discrimination of either model, and led to substantial miscalibration. Following recalibration the discrimination did not change, but good calibration for models incorporating the GRS was recovered. Comparing predictions between models with and without the GRS, 5% of participants or fewer changed their absolute risk by ±0.3% or more in either model. In summary, addition of a GRS did not meaningfully improve the performance of validated CRC-risk prediction models. At present, provision of genetic information is not useful for risk stratification for CRC.

## Background

Colorectal cancer (CRC) is a substantial global health burden^1^ and there is strong evidence that screening can reduce CRC mortality.^2–4^ The efficacy of screening programmes may be enhanced by targeting screening and screening intensity to those at greatest risk.^5^ Genome-wide association studies (GWAS) have identified over 40 independent loci unequivocally associated with the risk of CRC,^6^ and there is increasing interest in developing genetic risk scores (GRS) for a personalised risk assessment.^7^ To justify their use in clinical or population health practice, GRS must provide additional information over and above previously validated risk models.^8,9^ Here, using data from the UK Biobank, we examined the predictive value of a GRS for CRC either alone or in combination with validated CRC-risk models.

## Materials and methods

UK Biobank is a prospective cohort study of over 500,000 individuals^10^ of whom 488,377 are genotyped^11^ (Supplementary methods). Two of the best performing models (highest discrimination and good calibration) for the prediction of incident CRC,^12,13^ identified from a systematic review and external validation study^5^ were applied using data collected at baseline (Supplementary Table 1). Taylor et al.^13^ calculated predicted absolute risk by combining age-specific rates of CRC with estimated relative risks for different degrees of CRC family history. Wells et al.^12^ used a Cox regression model including age, diabetes, multi-vitamin usage, family history of colon cancer, years of education, body mass index, alcohol intake, physical activity, non-steroidal anti-inflammatory drug usage, red meat intake, smoking and oestrogen use (women only). Details of model calibration are presented in the supplementary methods. We constructed a weighted GRS as a linear combination of 41 autosomal single nucleotide polymorphisms (SNPs), with the allele dosage of each SNP multiplied by its associated log odds ratio from previously published GWAS studies (Supplementary Table 2).^6^

Model performance was evaluated in terms of calibration and discrimination. Calibration was visually assessed by plotting observed probability (calculated using the Kaplan–Meier estimator) against mean predicted probability by tenths of the predicted risk. We also assessed calibration of predicted relative risks by plotting the estimated hazard ratio (estimated using flexible parametric survival models) as a function of model-predicted hazard ratios (HR). Discrimination was assessed using the *C*-statistic (with 1 representing a perfect ability to discriminate between those who will subsequently develop the outcome of interest, and 0.5 representing no better ability than chance). We assessed the performance of (i) the predicted probabilities of the base models, (ii) the GRS alone and (iii) the two combined. As age can itself strongly contribute to model performance, we additionally assessed discrimination of both models after removing the effect of age. To ensure comparable calibration between the published models and the models augmented with the GRS, we also fitted flexible parametric survival models.^14^ We used two degree-of-freedom restricted cubic splines to model the baseline cumulative hazard of CRC, and included the overall predicted log HRs from the published models and the GRS as separate covariates. The fitted models were then used to predict 5-year absolute risks of CRC.

Participants with missing data on any of the required covariates were excluded from the analysis, which led to a different number of available participants for each model. We conducted a sensitivity analysis including only those participants who could be used in both models to ensure that estimates of model performance were directly comparable. In a second sensitivity analysis we removed related participants by identifying pairs of individuals who were first- or second-degree relatives (kinship coefficient greater than 0.08),^15,16^ and randomly dropping one member.

## Results

The number of available participants was 361,543 for the Taylor et al.^13^ model and 286,877 for the combined Wells et al.^12^ model (Supplementary Figure 1), comprising 1623 and 1294 CRC cases, respectively. Comparison between those included and excluded for each model showed broadly comparable characteristics (Supplementary Table 3).

The mean centred log GRS had a range of −2.022 to 2.411 and standard deviation of 0.495. It was weakly associated with self-reported family history of CRC, with a greater number of first degree relatives diagnosed with CRC associated with a higher GRS (Supplementary Table 4).

**Fig. 1:**
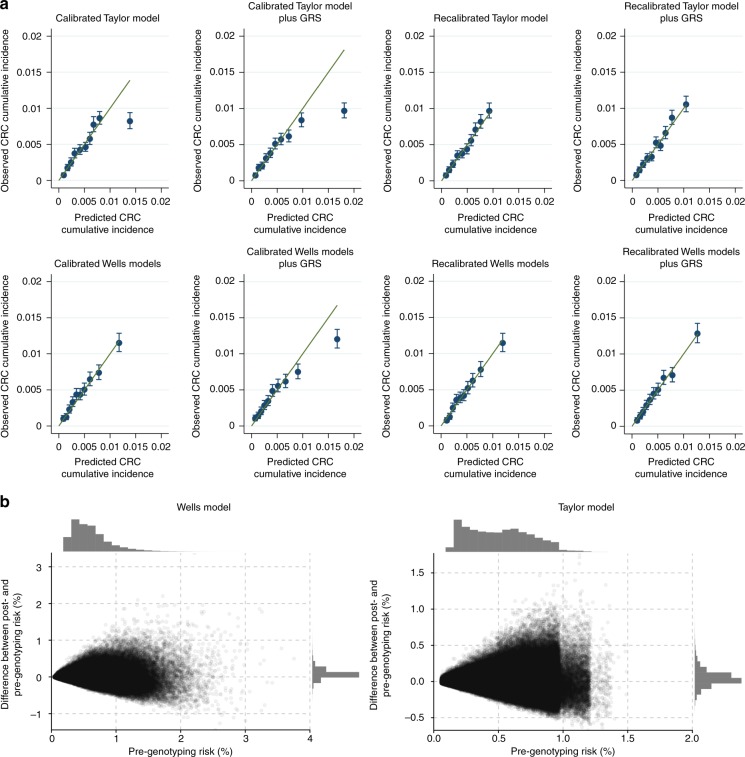
**a** Calibration plots for the Taylor et al.^13^ and Wells et al.^12^ models in the UK Biobank. The original models were initially calibrated to the UK Biobank population and following this the genetic risk score (GRS) was combined with the model’s original coefficient(s). To ensure comparable calibration between models with and without the GRS, we then further recalibrated by the predicted log hazard from the original model as a covariate in a flexible parametric survival model by itself, and with the addition of the GRS. **b** Change in the 5-year predicted probabilities (expressed as a percentage) of the recalibrated models after the addition of the genetic risk score. The x-axes are the predicted probabilities from the original models, and the *y*-axes are the difference in predicted probabilities between the GRS-augmented models and the original models. Histograms display the distribution of data along each axis. Note that the ranges of the axes differ between the two panels. The crowding of points close to the horizontal line at 0 on the *y*-axis illustrates that the addition of the GRS did not affect the predicted probabilities for the majority of participants

In the sample used for the Wells et al.^12^ model, the GRS alone provided modest discrimination for incident CRC (*C*-statistic 0.57, 95% CI: [0.55–0.58]), as it did for the sample used in the Taylor et al.^13^ model (0.56 [0.55–0.58]). This is greater than the discrimination afforded by the Taylor et al.^13^ model when excluding the effect of age (0.52 [0.51–0.53]), and comparable to that of the Wells et al.^12^ model after the age coefficient had been removed (0.58 [0.57–0.60]). The subsequent combination of the GRS with the original models did not improve discrimination (Wells et al.^12^ changed from 0.68 [0.67–0.69] to 0.69 [0.67–0.70], while for Taylor et al.^13^ it changed from 0.67 [0.65–0.68] to 0.67 [0.66–0.68], Supplementary Table 5), and resulted in poor calibration with substantial over-estimation of risk for those in the upper tenth of predicted risk (Fig. 1a and Supplementary Figure 2). This miscalibration was evident even when considering only relative risks, with the GRS both alone and in combination with the published models implying relative risks far more extreme than those observed in these data (Supplementary Figures 3 and 4). On recalibration by fitting the predicted log-hazard ratios and GRS as covariates in the models, calibration of the models including the GRS was vastly improved, and comparable with that of the models excluding the GRS (Fig. 1a). There was little difference in discrimination performance of models in participants with and without a family history of CRC (Supplementary Table 5).

The inclusion of the GRS in the recalibrated models did not result in a substantive change in the predicted probability for the majority of participants (Fig. 1b). For example, only 5% or fewer of participants had a change in predicted risk of 0.3% points or greater (Supplementary Table 6). Sensitivity analyses restricted to participants available for inclusion in both models, as well as further restricting to unrelated individuals, did not substantially affect the discrimination or calibration (Supplementary Tables 7 and 8, Supplementary Figures 5 and 6).

## Discussion

We examined the potential clinical utility of genetic information for CRC-risk prediction. In a large prospective cohort study, we showed that a GRS composed of 41 published, genome-wide significant SNPs for CRC, has poor discriminatory ability on its own and does not meaningfully improve model discrimination of established models, nor does it strongly influence the predicted probabilities for the vast majority of participants.

To our knowledge this is the first investigation of GRS-enhanced risk prediction models for CRC that has assessed both calibration and discrimination. Jeon et al.^7^ reported that a risk model including both genetic and environmental risk scores had slightly better discrimination than a model including an environmental risk score alone, but they could not assess model calibration. They also estimated individual recommended “starting ages” for screening, which differed by up to 12 years for men and 14 years for women. These estimates depend critically on the calibration of the model: any over- or under-estimation of risk will lead to more extreme variation in recommended starting ages, purely as an artefact of the miscalibration. We found that calibration of model-predicted probabilities deteriorated substantially with the inclusion of the GRS. This could have been due to inclusion of both the GRS and family history in the models, but we found that family history was only weakly associated with the GRS. Further, the GRS itself was miscalibrated, and implied relative risks that vastly overestimated the magnitude of the relative risks observed in our study. This is due to a phenomenon sometimes labelled as the “winner’s curse” or “statistical significance filter”, whereby estimates that surpass some threshold for significance tend to be overestimates of the underlying parameter. Our finding underlines the importance of careful recalibration of those GRSs based on SNPs selected as highly statistically significant in GWAS, and the potential for this to affect the performance of models, which do not assess or correct for it. This is particularly pertinent given that calibration is poorly reported in validation studies of risk prediction models and not commonly reported in GRS investigations, impairing the ability to assess the clinical usefulness of these models.

Although the inclusion of the GRS did not meaningfully improve model discrimination overall, and did not substantially change the predicted probabilities for the vast majority of participants (for example, 95% of participants had a change in probability of less than 0.3% points), provision of genetic information may have some utility in a two-step risk assessment. We found that the proportion of participants whose predicted risk increased or decreased by 0.3% points or more after inclusion of the GRS was much higher among those who had an initial risk of 1% or greater. While these numbers are only for illustration, they demonstrate that the added value of a GRS for risk prediction will be greater if it is applied to those at higher initial risk, rather than an entire population.

As larger studies are conducted more risk loci will likely be discovered, and more complete genetic information can potentially be incorporated into risk models. It is possible that the discrimination will improve beyond that already afforded by established risk models. On the other hand, as study sizes increase they will predominantly identify rare variants or variants that are more weakly associated with risk, so the potential for improvement in genetic prediction with the inclusion of these variants may be limited.

In summary, inclusion of a GRS did not improve the performance of two previously validated CRC-risk prediction models. Any practical benefit of using the GRS for CRC prediction is likely to only affect people already predicted to be at high risk based on existing models.

## Ethics approval and consent to participate

All participants provided written consent. UK Biobank has approval from the North West Multi-Centre Research Ethics Committee (MREC) and, in Scotland, the Community Health Index Advisory Group (CHIAG).

## Electronic supplementary material

Supplemental Material

## Data Availability

The UK Biobank is an open access resource. Researchers wishing to obtain data should apply directly to UK Biobank.

## References

[1] Ferlay J (2013). GLOBOCAN 2012v1.0, Cancer Incidence and Mortality Worldwide: IARC CancerBase No. 11 [Internet].

[2] Atkin Wendy, Wooldrage Kate, Parkin D Maxwell, Kralj-Hans Ines, MacRae Eilidh, Shah Urvi, Duffy Stephen, Cross Amanda J (2017). Long term effects of once-only flexible sigmoidoscopy screening after 17 years of follow-up: the UK Flexible Sigmoidoscopy Screening randomised controlled trial. The Lancet.

[3] Lin JS (2016). U.S. Preventive Services Task Force Evidence Syntheses, formerly Systematic Evidence Reviews. Screening for Colorectal Cancer: A Systematic Review for the US Preventive Services Task Force.

[4] Brenner H., Stock C., Hoffmeister M. (2014). Effect of screening sigmoidoscopy and screening colonoscopy on colorectal cancer incidence and mortality: systematic review and meta-analysis of randomised controlled trials and observational studies. BMJ.

[5] Smith Todd, Muller David C, Moons Karel G M, Cross Amanda J, Johansson Mattias, Ferrari Pietro, Fagherazzi Guy, Peeters Petra H M, Severi Gianluca, Hüsing Anika, Kaaks Rudolf, Tjonneland Anne, Olsen Anja, Overvad Kim, Bonet Catalina, Rodriguez-Barranco Miguel, Huerta Jose Maria, Barricarte Gurrea Aurelio, Bradbury Kathryn E, Trichopoulou Antonia, Bamia Christina, Orfanos Philippos, Palli Domenico, Pala Valeria, Vineis Paolo, Bueno-de-Mesquita Bas, Ohlsson Bodil, Harlid Sophia, Van Guelpen Bethany, Skeie Guri, Weiderpass Elisabete, Jenab Mazda, Murphy Neil, Riboli Elio, Gunter Marc J, Aleksandrova Krasimira Jekova, Tzoulaki Ioanna (2018). Comparison of prognostic models to predict the occurrence of colorectal cancer in asymptomatic individuals: a systematic literature review and external validation in the EPIC and UK Biobank prospective cohort studies. Gut.

[6] Peters U, Bien S, Zubair N (2015). Genetic architecture of colorectal cancer. Gut.

[7] Jeon, J. et al. Determining risk of colorectal cancer and starting age of screening based on lifestyle, environmental, and genetic factors. *Gastroenterology*. **154**, 2152–2164 (2018). 10.1053/j.gastro.2018.02.021 PMC5985207 29458155

[8] Ioannidis JA, Tzoulaki I (2010). What makes a good predictor? The evidence applied to coronary artery calcium score. J. Am. Med. Assoc..

[9] Tzoulaki I, Liberopoulos G, Ioannidis JP (2009). Assessment of claims of improved prediction beyond the Framingham risk score. J.Am. Med. Assoc..

[10] Sudlow C (2015). UK Biobank: an open access resource for identifying the causes of a wide range of complex diseases of middle and old age. PLoS Med..

[11] Bycroft C., et al. Genome-wide genetic data on ~500,000 UK Biobank participants. *bioRxiv*. 2017. 10.1101/166298.

[12] Wells BJ, Kattan MW, Cooper GS, Jackson L, Koroukian S (2014). Colorectal cancer predicted risk online (CRC-PRO) calculator using data from the multi-ethnic cohort study. J. Am. Board. Fam. Med..

[13] Taylor DP (2011). How well does family history predict who will get colorectal cancer? Implications for cancer screening and counseling. Genet. Med..

[14] Lambert, P. STPM2: Stata module to estimate flexible parametric survival models. Statistical Software Components S457128. (Boston College Department of Economics, 2010).

[15] Pazoki R (2018). Genetic predisposition to high blood pressure and lifestyle factors: associations with midlife blood pressure levels and cardiovascular events. Circulation.

[16] Manichaikul A (2010). Robust relationship inference in genome-wide association studies. Bioinformatics.

