# Supplementary material for: The added value of genetic information in colorectal cancer risk prediction models: development and evaluation in the UK Biobank prospective cohort study
Source: Br J Cancer. 2018 Oct 16;119(8):1036–9. doi: 10.1038/s41416-018-0282-8 (PMC6203780; doi:10.1038/s41416-018-0282-8)
Supplement: Supplementary file 1 — Supplemental Material [file 41416_2018_282_MOESM1_ESM.pdf]

The added value of genetic information in colorectal cancer risk prediction models

Todd Smith, Marc J Gunter, Ioanna Tzoulaki, David C Muller

Online-only Supplement Table of Contents

Title	Page Number
Supplementary Methods	2
Supplementary Table 1	3
Supplementary Table 2	7
Supplementary Table 3	8
Supplementary Table 4	9
Supplementary Table 5	10
Supplementary Table 6	11
Supplementary Table 7	12
Supplementary Table 8	13
Supplementary Figure 1	14
Supplementary Figure 2	15
Supplementary Figure 3	16
Supplementary Figure 4	17
Supplementary Figure 5	18
Supplementary Figure 6	19

Supplementary Methods

UK Biobank Study Sample

In brief over 500,000 participants were recruited from National Health Service registers and subsequently assessed at 22 centres throughout the UK between 2006 and 2010. They are followed-up for cancer incidence and death via linkage to population registries. Further details of recruitment and data collection have been described in detail previously [1-3]. Participants were followed up from the date of baseline attendance until the date of diagnosis of an invasive primary cancer (excluding non-melanoma skin cancer), date of death, or January 1, 2015, whichever occurred first. CRC was defined by the International Statistical Classification of Diseases and Related Health Problems 10th Revision codes: C18 (except C18.1, Appendix), C19 and C20. For the purposes of this study we only included participants who identified as being of white ethnicity since published GWAS have been overwhelmingly conducted with participants of European descent.

Genotyping and Imputation

Details of genotyping and imputation within the UK Biobank have already been published [4]. In summary, participants were genotyped using the UK BiLEVE Axiom TM and UK Biobank Axiom TM arrays. Imputation to ~92 million markers was subsequently carried out using the Haplotype Reference Consortium.

Estimation of absolute risk

In both the Taylor et al. and Wells et al. models, calibrated absolute risks were calculated by combining estimated rates or risks with predicted relative risks or hazard ratios based on the published models. In the Taylor et al. [5] model, we calculated 5-year age-specific rates for both CRC and the competing event of death using data from available participants in the UK Biobank, and combined these rates with the published familial relative risks to estimate the 5 year absolute risk of CRC. In Wells et al.'s [6] model, we calculated the marginal/population level 5-year survival function for CRC among available participants in UK Biobank, and combined this with the model predicted hazard ratios to obtain individual level estimates of the survival function. The Wells et al. model did not account for the competing risk of death.

Statistical Software

Stata version 13 software was used for all analyses.

Supplementary Table 1: Variable construction in the UK Biobank

	Variable construction overview	Number of ineligible participants due to incomplete or missing data, or who were outside the modelled age range (initial sample 433,899)
Taylor et al. [5]		
Age	40 - 70 years old.<br>Variable used:<br>*Age at recruitment (21022)*	11
Family history of colorectal cancer	Variables provided reported history of "bowel cancer" for parents and siblings (allowing up to a maximum of 3 cases). This was taken to represent colorectal cancer.<br>Variables used:<br>*Number of full brothers (1873), Number of full sisters (1883), Father still alive (1797), Mother still alive (1835), Illnesses of siblings (20111), Illnesses of father (20107), Illnesses of mother (20110)*	72,345
Wells et al. [6]		
Age	40 -70 years old.<br>Variable used:<br>*Age at recruitment (21022)*	11
Diabetes	Variable provided information on the diagnosis of diabetes.<br>Variable used:<br>*Diabetes diagnosed by doctor (2443)*	989
Multi-vitamin	Variable detailed regular use of different vitamins and minerals. The "Multivitamins +/- minerals" response was taken to represent regular use of multivitamins.<br>Variable used:<br>*Vitamin and mineral supplements (6155)*	2,153
Family History of Colon Cancer	Variables provided reported history of "bowel cancer" for parents and siblings (allowing up to a maximum of 3 cases). As no distinction could be made between colon and rectal cancer all were assumed to be colon.<br>Variables used:<br>*Number of full brothers (1873), Number of full sisters (1883), Father still alive (1797), Mother still alive (1835), Illnesses of siblings (20111), Illnesses of father (20107), Illnesses of mother (20110)*	72,345

	Variable construction overview (continued)	Number of ineligible participants due to incomplete or missing data, or who were outside the modelled age range (initial sample 433,899) (continued)
Years of Education	Variables provided 1) qualifications held by the participants and 2) age at which participants completed continuous full time education (for all bar those who had a College or University Degree, in which case this was omitted).<br>To calculate the number of years of education in all participants, those with a Degree were set to have completed full time education at age 21. A school starting age of 5 was then deducted from all to obtain the total number of years of education. Those who reported not going to school or who had a negative number of years of schooling were set to 0. A maximum number of years of education of 20 was used, in line with the online version of the model [a], any values above this were set to 20.<br>Variables used:<br>*Age completed full time education (845)*<br>*Qualifications (6138)*	5,447
Body Mass Index	Variable provided a body mass index constructed from measured height and weight. A maximum BMI of 34.8 kg/m^2 was used, in line with the online model [a], any values above this were set to 34.8.<br>Variables used:<br>*Body mass index (21001)*	1,401
Alcoholic drinks per day	Variables detailed alcohol intake frequency and, based on this, a weekly or monthly measure of beer/cider, champagne/white wine, red wine, fortified wine and spirits intake. A maximum of 12 drinks per day was used, in line with the online model [a], any values above this were set to 12.<br>Variables used:<br>*Alcohol intake frequency (1558), Average weekly: beer plus cider intake (1588), champagne plus white wine intake (1578), fortified wine intake (1608), red wine intake (1568) and spirits intake (1598). Average monthly: beer plus cider intake (4429), champagne plus white wine intake (4418), fortified wine intake (4451), red wine intake (4407) and spirits intake (4440).*	68,892

	Variable construction overview (continued)	Number of ineligible participants due to incomplete or missing data, or who were outside the modelled age range (initial sample 433,899) (continued)
Hours of moderate and strenuous activity per day (male only)	The model's description was "hours of moderate physical activity per day" though the description of the data in the derivation cohort was "hours of moderate or strenuous activity per day" so the later was used. Variables detailed the number of days per typical week that moderate and vigorous activity was undertaken (for ten minutes or more) as well as the number of minutes per day this was undertaken on a typical day (if the answer to the preceding question was 1 or greater). Any duration less than 10 minutes was set to 0 and the number of hours per week calculated, following the International Physical Activity Questionnaire Guidelines [b]. A maximum value of 4 hours per day was used, in line with the online model [a], any values above this were set to 4.<br>Variables used:<br>*Number of days/week of moderate physical activity 10+ minutes (884), Duration of moderate activity (894), Number of days/week of vigorous physical activity 10+ minutes (904), Duration of vigorous activity (914)*	12,320
Regular use of aspirin/NSAIDs	For men the variable was "aspirin" while for women it was "NSAIDS".<br>Variables detailed the regular use of aspirin and or ibuprofen. The aspirin response was used for men and both were combined to represent NSAIDs for women.<br>Variable used:<br>*Medication for pain relief, constipation, heartburn (6154)*	4,776
Ounces of red meat intake per day (male only)	Variables provided details of Beef, Lamb/mutton, Pork and Processed meat intake per week (never, <1/week, 1/week, 2-4/week, 5-6/week, ≥1 daily), these were adjusted to provide absolute values of 0, 0.5, 1, 3, 5.5 and 7 per week respectively. These were then summed and adjusted to provide ounces per day (based on 4 ounces per intake). A maximum value of 5 ounces per day was used, in line with the online model [a], any values above this were set to 5.<br>Variables used:<br>*Beef intake (1369), Lamb/mutton intake (1379), Pork intake (1389), Processed meat intake (1349).*	2,270
Pack years of smoking	The construction of this variable required the use of multiple variables based on subjects smoking habits at recruitment and prior to it. A maximum value of 50 was used, in line with the online model [a], any values above this were set to 50.<br>Variables used:<br>*Multiple variables from category 100058*	10,514

	Variable construction overview (continued)	Number of ineligible participants due to incomplete or missing data, or who were outside the modelled age range (initial sample 433,899) (continued)
Have you used estrogen (female only)	The construction of this variable required the use of variables detailing hormone replacement therapy use and oral contraceptive pill use (it was assumed that all oral contraceptives contained estrogen). Variables used: *Ever taken oral contraceptive pill (2784), Age when last used oral contraceptive pill (2804), Ever used hormone-replacement therapy (HRT) (2814), Age last used hormone replacement therapy (HRT) (3546).*	1,609

[a] Online model (http://riskcalc.org/ColorectalCancer/). [b] International Physical Activity Questionnaire [7]

Supplementary Table 2: Single nucleotide polymorphisms associated with colorectal cancer from Peters et al. [8]

Single Nucleotide Polymorphism	Odds Ratio	Genotyped in UKB	Imputation Quality Measure
rs10911251	1.09		0.998
rs6687758	1.09	■	1
rs11903757	1.16		0.997
rs812481	1.09		0.989
rs35360328	1.14		0.990
rs10936599	1.04	■	1
rs647161	1.06	■	0.990
rs1321311	1.10	■	1
rs7758229	1.28	■	1
rs16892766	1.27	■	1
rs6983267	1.19	■	1
rs719725	1.14		0.999
rs10795668	1.12	■	1
rs704017	1.10	■	1
rs1035209	1.12	■	0.998
rs12241008	1.19		0.997
rs11196172	1.14		0.989
rs1535	1.09	■	1
rs3824999	1.08	■	1
rs3802842	1.11	■	1
rs10849432	1.14	■	1
rs3217810	1.10		0.903
rs10774214	1.17		0.995
rs11169552	1.09	■	1
rs7136702	1.06	■	1
rs3184504	1.09	■	1
rs73208120	1.16		0.969
rs1957636	1.08	■	0.999
rs4444235	1.11	■	1
rs4779584	1.35	■	1
rs9929218	1.10	■	1
rs12603526	1.10	■	1
rs4939827	1.20	■	1
rs7229639	1.22		0.983
rs10411210	1.15	■	1
rs1800469	1.09		0.999
rs2423279	1.10		0.999
rs4813802	1.10	■	0.987
rs961253	1.12	■	1
rs6066825	1.09		0.989
rs4925386	1.08	■	1

Further details on genotyping and imputation in the UK Biobank have been published elsewhere [4]

Supplementary Table 3: Comparison of eligible and ineligible participants in each model

	Eligible UK Biobank Cohort	Taylor et al. [5]		Wells et al. [6]	
		Eligible	Ineligible	Eligible	Ineligible
Number of Participants (% of eligible cohort)	433,899	361, 543 (83)	72,356 (17)	286,877 (66)	147,022 (34)
Male (%)	200,497 (46)	161,965 (45)	38,532 (53)	132,637 (46)	67,860 (46)
Female (%)	233, 402 (54)	199, 578 (55)	33,824 (47)	154,240 (54)	79,162 (54)
Colorectal Cancer Cases [a] (C.I.R./100,000 years)	2,007 (96)	1,623 (93)	384 (110)	1,294 (93)	713 (100)
Follow-up time (person years)	2,100,144	1,751,445	348,698	1,388,191	711,953
Time to diagnosis (years) for cases (I.Q.R)[c]	2.6 (1.3-3.8)	2.6 (1.3-3.8)	2.6 (1.4-3.7)	2.6 (1.4-3.8)	2.5 (1.3-3.7)
Age					
% with no data [b]	0	0	0	0	0
Of those with data (I.Q.R.) [c]	58 (50-63)	57 (50-63)	60 (52-64)	57 (50-63)	59 (51-64)
Parental or sibling history of bowel cancer					
% with incomplete data [b]	17	0	100	0	49
Of those with data					
% with	12	12	9	12	12
% without	88	88	91	88	88
BMI kg/m^2					
% with no data [b]	0	0	0	0	1
Of those with data (I.Q.R.) [c]	27 (24-30)	27 (24-30)	27 (25-31)	26 (24-29)	27 (25-31)
Smoking status					
% with no data [b]	0	0	1	0	1
Of those with data					
% Never	54	55	48	55	52
% Previous	35	35	38	35	35
% Current	11	10	14	9	13
Alcohol status					
% with no data [b]	0	0	0	0	0
Of those with data					
% Never	3	3	4	4	3
% Previous	3	3	5	4	3
% Current	93	94	91	93	94

	Eligible UK Biobank Cohort	Taylor et al. [5]		Wells et al. [6]	
		Eligible (continued)	Ineligible (continued)	Eligible (continued)	Ineligible (continued)
Days/week of vigorous physical activity					
% with no data [b]	5	4	9	2	10
Of those with data (I.Q.R.) [c]	1 (0-3)	1 (0-3)	1 (0-3)	1 (0-3)	1 (0-3)
Processed meat consumption					
% with no data [b]	0	0	0	0	0
Of those with data					
% Never	9	9	8	9	9
% <1 / week	30	31	28	30	30
% 1 / week	30	30	29	30	29
% 2-4 / week	28	27	30	28	28
% 5-6 / week	3	3	4	3	3
% ≥ 1 / day	1	1	1	1	1

[a] cancer site was defined by the International Statistical Classification of Disease and Related Health Problems, 10th Revision, colorectal encompassed C18-C20 (excluding C181, appendix). [b] % with no data/incomplete data, encompasses missing values and the responses “do not know” and “prefer not to answer”. [c] median values with interquartile range (I.Q.R.). Cohort assessed over a 5 year time horizon from recruitment. Percentages are rounded to the nearest whole percentage point.

Supplementary Table 4: Association between family history of colorectal cancer and log GRS. Estimates are from linear regression models of log-GRS on family history, as a continuous and a categorical variable

log GRS variable construction (y)	Family History variable construction (x)	Regression coefficient	95% confidence interval
continuous	continuous	0.041	[0.036, 0.046]
continuous	Categorical		
	No family history	Reference	
	1 relative	0.042	[0.037, 0.048]
	2 relatives	0.073	[0.053, 0.093]
	3 relatives	0.094	[-0.015, 0.203]

The mean centred log GRS ranged from -2.022 to 2.411 with a standard deviation of 0.495. Family History of Bowel Cancer ranged between 0-3 first degree relatives. Regression coefficient was determined in those eligible for inclusion in the Taylor et al. [5] analysis (n = 361,543), within which Wells et al. [6] was nested.

Supplementary Table 5: C-statistics [95% confidence intervals] for the original model, GRS, and original model augmented with GRS, both overall and by family history of colorectal cancer

	Taylor et al [5].			Wells et al. [6], [a]		
	All participants (N = 361,543, N_{cases}=1,623)	No family history (N = 319,185, N_{cases} = 1,368)	With family history (N = 42,358, N_{cases} = 255)	All participants (N = 286,877 N_{cases} = 1,294)	No family history (N = 253,324 , N_{cases} = 1,081)	With family history (N = 33,553 , N_{cases} = 213)
Original Model	0.67 [0.65 to 0.68]	0.67 [0.66 to 0.69]	0.64 [0.61 to 0.67]	0.68 [0.67 to 0.69]	0.68 [0.66 to 0.69]	0.67 [0.63 to 0.71]
Original model, excluding age [b]	0.52 [0.51 to 0.53]	0.50 [0.50 to 0.50]	0.50 [0.49 to 0.52]	0.58 [0.57 to 0.60]	0.57 [0.55 to 0.59]	0.60 [0.56 to 0.64]
GRS alone [c]	0.56 [0.55 to 0.58]	0.57 [0.55 to 0.58]	0.55 [0.52 to 0.59]	0.57 [0.55 to 0.58]	0.56 [0.55 to 0.58]	0.57 [0.53 to 0.60]
Original Model + GRS [d]	0.67 [0.66 to 0.68]	0.68 [0.66 to 0.69]	0.64 [0.61 to 0.67]	0.69 [0.67 to 0.70]	0.69 [0.67 to 0.70]	0.68 [0.64 to 0.71]
Original Model (fully recalibrated) [e]	0.67 [0.66 to 0.68]	0.67 [0.66 to 0.69]	0.65 [0.61 to 0.68]	0.68 [0.67 to 0.70]	0.68 [0.67 to 0.70]	0.67 [0.63 to 0.71]
Original Model + GRS (fully recalibrated) [f]	0.68 [0.67 to 0.70]	0.69 [0.67 to 0.70]	0.66 [0.62 to 0.69]	0.69 [0.68 to 0.71]	0.69 [0.68 to 0.71]	0.69 [0.65 to 0.72]

Abbreviations: GRS, genetic risk score. [a] The results of the individual Wells et al. [6] male and female models were merged and the discrimination estimated. [b] The discrimination of the hazard ratio used by the model is assessed independently of the model's construction, to remove the effect of age the age coefficients were omitted from the Wells model. [c] The GRS was used directly as the scoring rule [d] log GRS was combined with the predicted log hazard ratio from the original models. [e] The predicted log hazard ratio from the original model was fitted as a covariate in a flexible parametric survival model in order to better recalibrate the predicted probabilities. [f] The predicted log hazard ratio from the original model and the log GRS were fitted as covariates in a flexible parametric survival models in order to better recalibrate the predicted probabilities. The UK Biobank participants were asked about a family history of "bowel cancer" in parents and siblings, this was taken to be synonymous with colorectal cancer. Number of cases is within a 5 year time horizon from recruitment.

Supplementary Table 6: Change in absolute risk after the addition of the GRS in the recalibrated models

	Taylor et al. [5]			Wells et al. [6]		
	Initial Absolute Risk			Initial Absolute Risk		
	<1%	≥1%	Overall	<1%	≥1%	Overall
Number of Individuals	356,219	5,324	361,543	266,526	20,351	286,877
Number with a change in absolute risk ≥ ± 0.1% (% of that initial absolute risk group)	108,773 (31)	3,773(71)	112,546 (31)	73,707 (28)	15,267 (75)	88,974 (31)
Number with a change in absolute risk ≥ ±0.2% (% of that initial absolute risk group)	36,352 (10)	2,377 (45)	38,729 (11)	22,135 (8)	10,514 (52)	32,649 (11)
Number with a change in absolute risk ≥± 0.3% (% of that initial absolute risk group)	12,017 (3)	1,334 (25)	13,351 (4)	7,170 (3)	6,642 (33)	13,812 (5)

Supplementary Table 7: C-statistics [95% confidence intervals] for the original model, GRS, and original model augmented with GRS, both overall and by family history of colorectal cancer, restricting the analysis to participants available for both the Taylor et al and Wells et al models

	Taylor et al [5].			Wells et al. [6], [a]		
	All participants	No family history	With family history	All participants	No family history	With family history
	(N = 286,877, N_{cases}= 1,294)	(N = 253,324 , N_{cases} = 1,081)	(N = 33,553 , N_{cases} = 213)	(N = 286,877 , N_{cases} = 1,294)	(N = 253,324 , N_{cases} = 1,081)	(N = 33,553 , N_{cases} = 213)
Original Model	0.66 [0.65, 0.68]	0.67 [0.65, 0.68]	0.65 [0.61, 0.68]	0.68 [0.67 to 0.69]	0.68 [0.66 to 0.69]	0.67 [0.63 to 0.71]
Original model Hazard Ratio alone, excluding age [b]	0.52 [0.51, 0.53]	0.50 [0.50, 0.50]	0.50 [0.49, 0.52]	0.58 [0.57 to 0.60]	0.57 [0.55 to 0.59]	0.60 [0.56 to 0.64]
GRS alone [c]	0.57 [0.55, 0.58]	0.56 [0.55, 0.58]	0.57 [0.53, 0.60]	0.57 [0.55 to 0.58]	0.56 [0.55 to 0.58]	0.57 [0.53 to 0.60]
Original Model + GRS [d]	0.67 [0.66, 0.68]	0.67 [0.66, 0.69]	0.65 [0.62, 0.68]	0.69 [0.67 to 0.70]	0.69 [0.67 to 0.70]	0.68 [0.64 to 0.71]
Original Model (fully recalibrated) [e]	0.67 [0.66, 0.68]	0.67 [0.65, 0.68]	0.65 [0.61, 0.68]	0.68 [0.67 to 0.70]	0.68 [0.67 to 0.70]	0.67 [0.63 to 0.71]
Original Model + GRS (fully recalibrated) [f]	0.68 [0.67, 0.70]	0.68 [0.67, 0.70]	0.66 [0.63, 0.70]	0.69 [0.68 to 0.71]	0.69 [0.68 to 0.71]	0.69 [0.65 to 0.72]

Abbreviations: GRS, genetic risk score. [a] The results of the individual Wells et al. [6] male and female models were merged and the discrimination estimated. [b] The discrimination of the hazard ratio used by the model is assessed independently of the model's construction, to remove the effect of age the age coefficients were omitted from the Wells model. [c] The GRS was used directly as the scoring rule [d] log GRS was combined with the predicted log hazard ratio from the original models. [e] The predicted log hazard ratio from the original model was fitted as a covariate in a flexible parametric survival model in order to better recalibrate the predicted probabilities. [f] The predicted log hazard ratio from the original model and the log GRS were fitted as covariates in a flexible parametric survival models in order to better recalibrate the predicted probabilities. The UK Biobank participants were asked about a family history of "bowel cancer" in parents and siblings, this was taken to be synonymous with colorectal cancer. Number of cases is within a 5 year time horizon from recruitment.

Supplementary Table 8: C-statistics [95% confidence intervals] for the original model, GRS, and original model augmented with GRS, both overall and by family history of colorectal cancer restricting the analysis to participants available for both the Taylor et al and Wells et al models, and excluding 1st and 2nd degree relatives

	Taylor et al [5].			Wells et al. [6], [a]		
	All participants (N = 274,708, N_{cases}= 1,235)	No family history (N = 242,577, N_{cases} = 1,032)	With family history (N = 32,131, N_{cases} = 203)	All participants (N = 274,708, N_{cases} = 1,235)	No family history (N = 242,577, N_{cases} = 1,032)	With family history (N = 32,131, N_{cases} = 203)
Original Model	0.66 [0.65, 0.68]	0.67 [0.65, 0.68]	0.64 [0.61, 0.68]	0.68 [0.67, 0.69]	0.68 [0.66, 0.69]	0.67 [0.63, 0.71]
Original model Hazard Ratio alone, excluding age [b]	0.52 [0.51, 0.53]	0.50 [0.50, 0.50]	0.50 [0.49, 0.52]	0.58 [0.57, 0.60]	0.57 [0.55, 0.59]	0.60 [0.56, 0.64]
GRS alone [c]	0.56 [0.55, 0.58]	0.56 [0.54, 0.58]	0.57 [0.53, 0.61]	0.56 [0.55, 0.58]	0.56 [0.54, 0.58]	0.57 [0.53, 0.61]
Original Model + GRS [d]	0.67 [0.65, 0.68]	0.67 [0.65, 0.68]	0.65 [0.62, 0.69]	0.69 [0.67, 0.70]	0.68 [0.67, 0.70]	0.68 [0.64, 0.71]
Original Model (fully recalibrated) [e]	0.67 [0.66, 0.68]	0.67 [0.65, 0.68]	0.65 [0.61, 0.68]	0.68 [0.67, 0.70]	0.68 [0.67, 0.70]	0.67 [0.63, 0.71]
Original Model + GRS (fully recalibrated) [f]	0.68 [0.67, 0.70]	0.68 [0.67, 0.70]	0.66 [0.63, 0.70]	0.69 [0.68, 0.71]	0.69 [0.68, 0.71]	0.68 [0.65, 0.72]

Abbreviations: GRS, genetic risk score. [a] The results of the individual Wells et al. [6] male and female models were merged and the discrimination estimated. [b] The discrimination of the hazard ratio used by the model is assessed independently of the model's construction, to remove the effect of age the age coefficients were omitted from the Wells model. [c] The GRS was used directly as the scoring rule [d] log GRS was combined with the predicted log hazard ratio from the original models. [e] The predicted log hazard ratio from the original model was fitted as a covariate in a flexible parametric survival model in order to better recalibrate the predicted probabilities. [f] The predicted log hazard ratio from the original model and the log GRS were fitted as covariates in a flexible parametric survival models in order to better recalibrate the predicted probabilities. The UK Biobank participants were asked about a family history of "bowel cancer" in parents and siblings, this was taken to be synonymous with colorectal cancer. Number of cases is within a 5 year time horizon from recruitment.

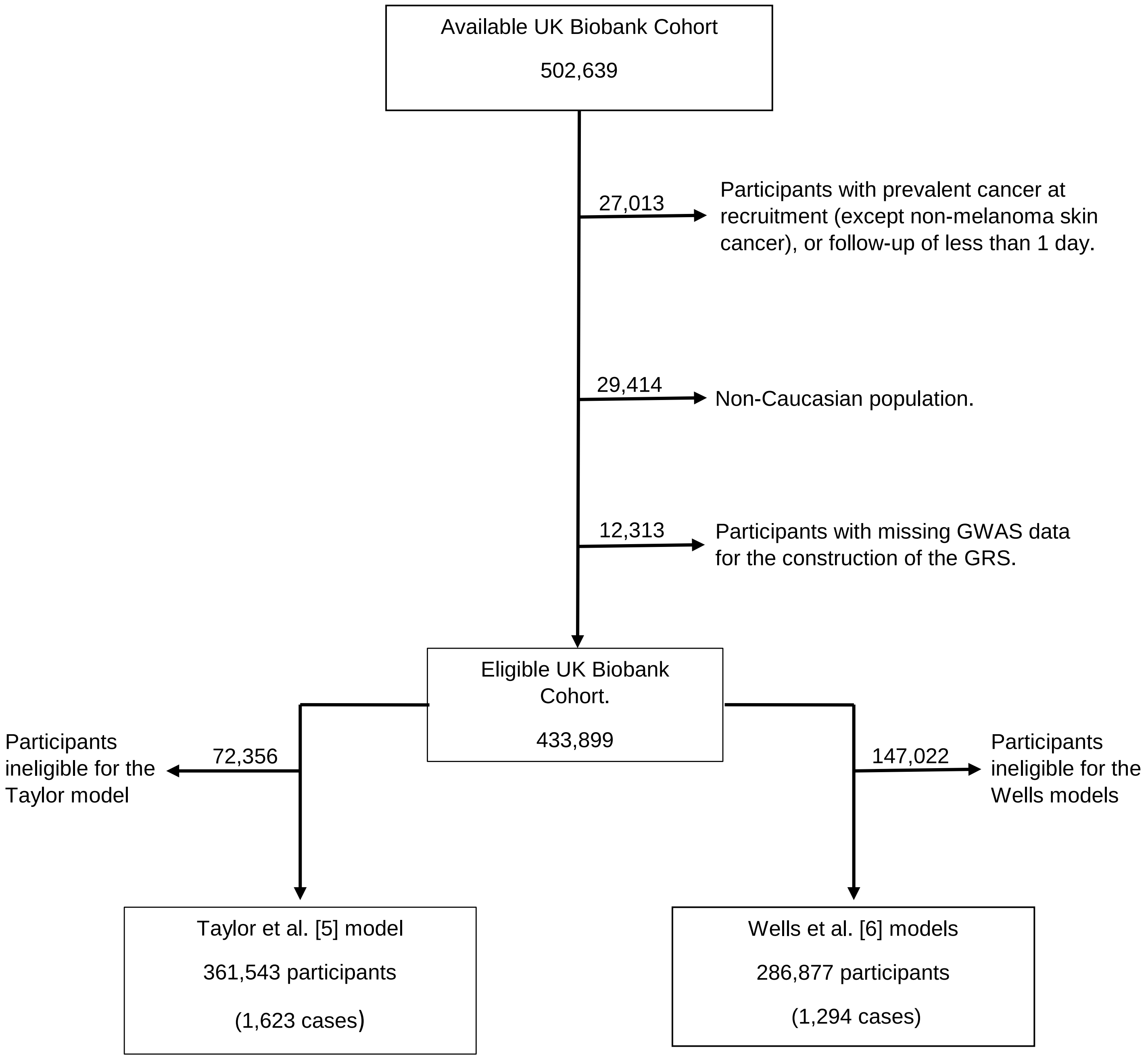

Supplementary Figure 1: Flow chart of participant eligibility criteria

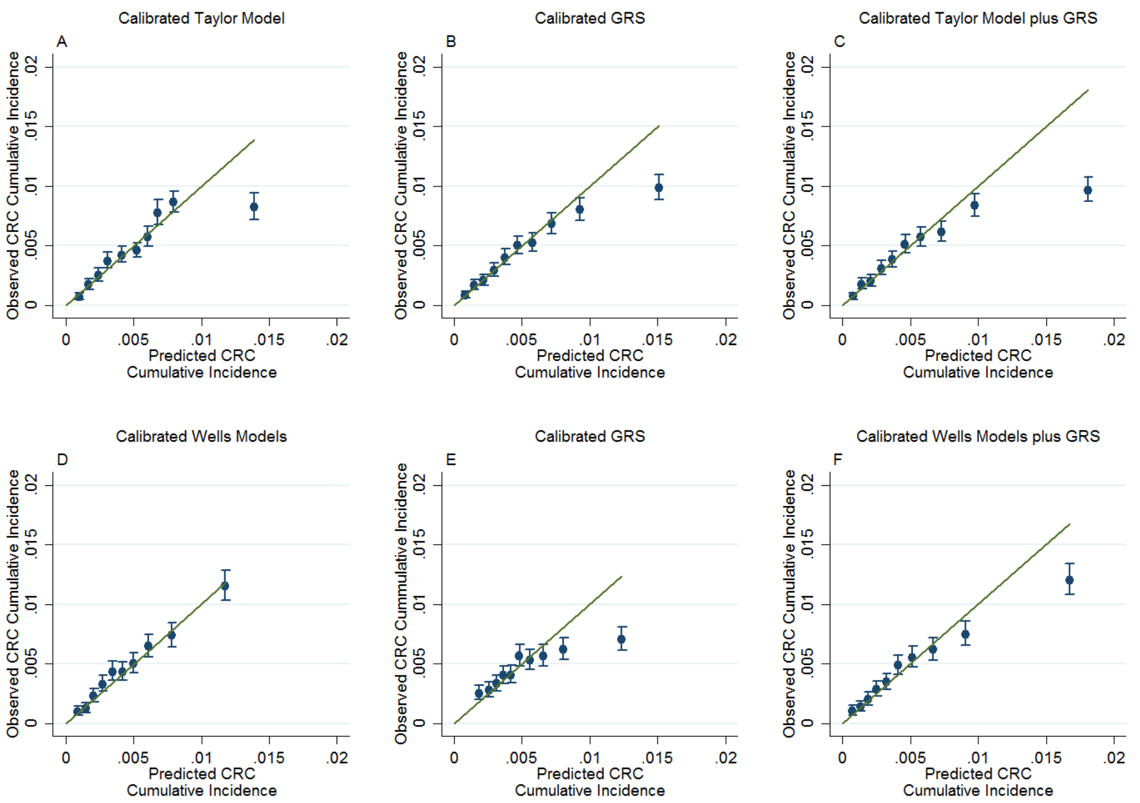

Supplementary Figure 2: Calibration plots for the Taylor et al. [5] and Wells et al. [6] models in the UK Biobank. The original models were initially calibrated to the UK Biobank population (inset A and D). Following this the genetic risk score (GRS) was substituted for the model's original coefficient(s) (inset B and E) as well as combined with it (inset C and F).

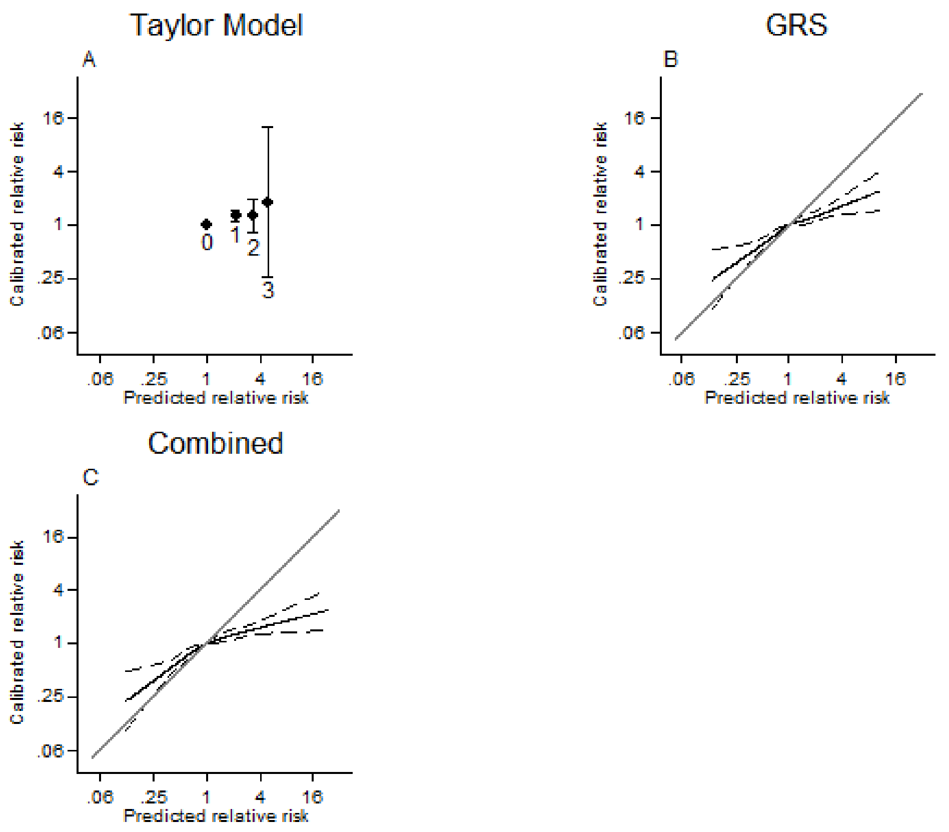

Supplementary Figure 3: Relative risk calibration plots for the Taylor et al. [5] model. Estimated relative risks in the UK Biobank as a function of (A) the predicted relative risk from the original model (numbers on the plot represent number of first degree relatives with bowel cancer), (B) the genetic risk score (GRS), and (C) the combination of the two. The estimated/calibrated relative risks are hazard ratios estimated by modelling the hazard of CRC as a function of the log predicted relative risks using flexible parametric survival models. The solid black line represents the estimate and the dashed lines the 95% confidence interval. The solid grey line is a line of equivalence.

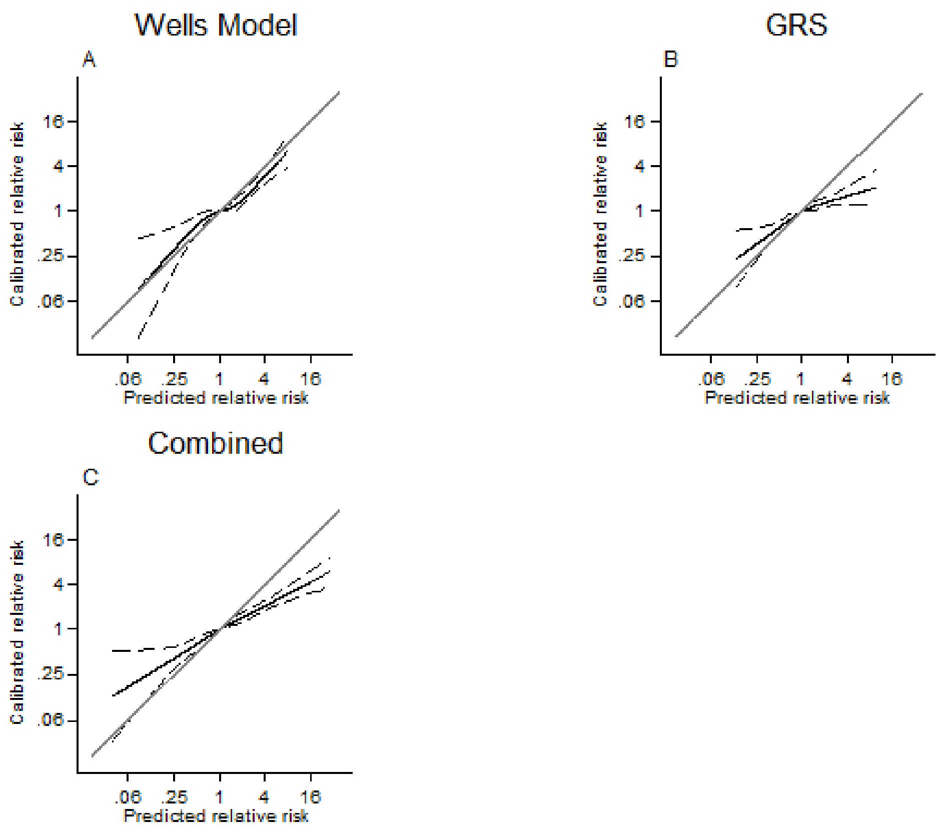

Supplementary Figure 4: Relative risk calibration plots for the Wells et al. [6] model. Estimated relative risks in the UK Biobank as a function of (A) the predicted relative risk from the original model, (B) the genetic risk score (GRS), and (C) the combination of the two. The estimated/calibrated relative risks are hazard ratios estimated by modelling the hazard of CRC as a function of the log predicted relative risks using flexible parametric survival models. The solid black line represents the estimate and the dashed lines the 95% confidence interval. The solid grey line is a line of equivalence.

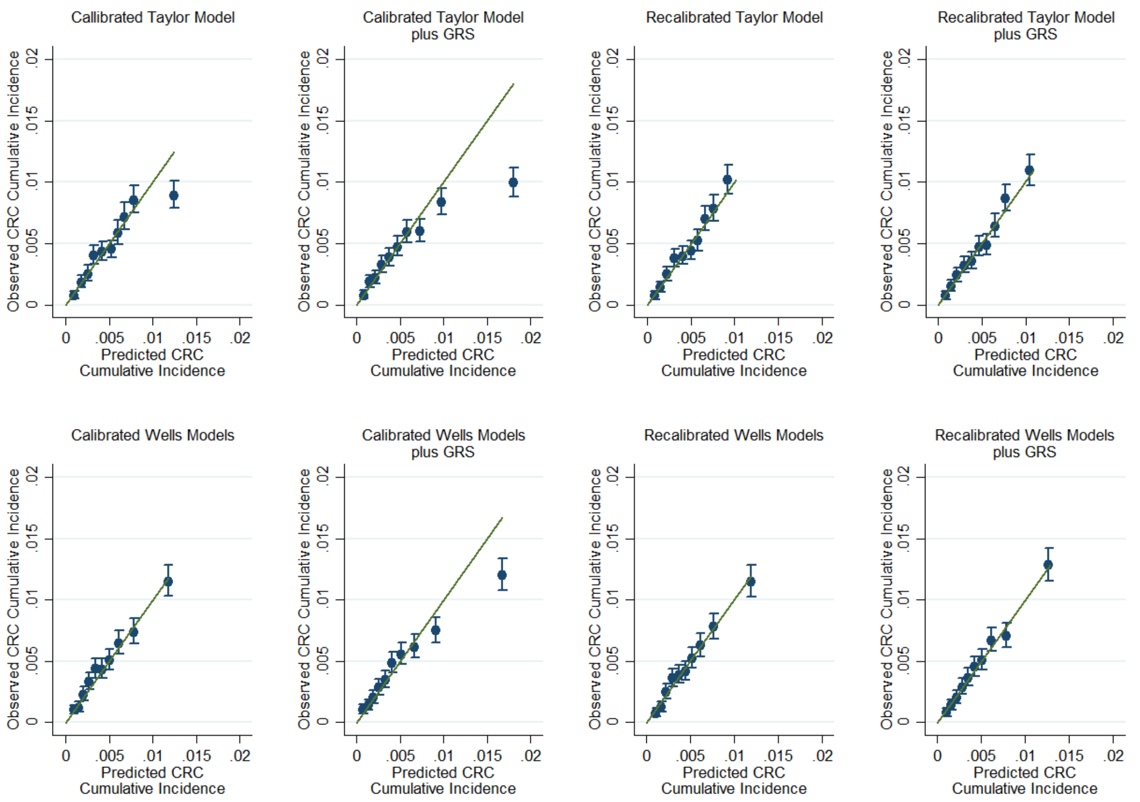

Supplementary Figure 5: Calibration plots for the Taylor et al. [5] and Wells et al. [6] models in the UK Biobank, restricting the analysis to participants available for both the Taylor et al. and Wells et al. models. The original models were initially calibrated to the UK Biobank population and following this the genetic risk score (GRS) was combined with the model's original coefficient(s). To ensure comparable calibration between models with and without the GRS, we then further recalibrated by the predicted log hazard from the original model as a covariate in a flexible parametric survival model by itself, and with the addition of the GRS.

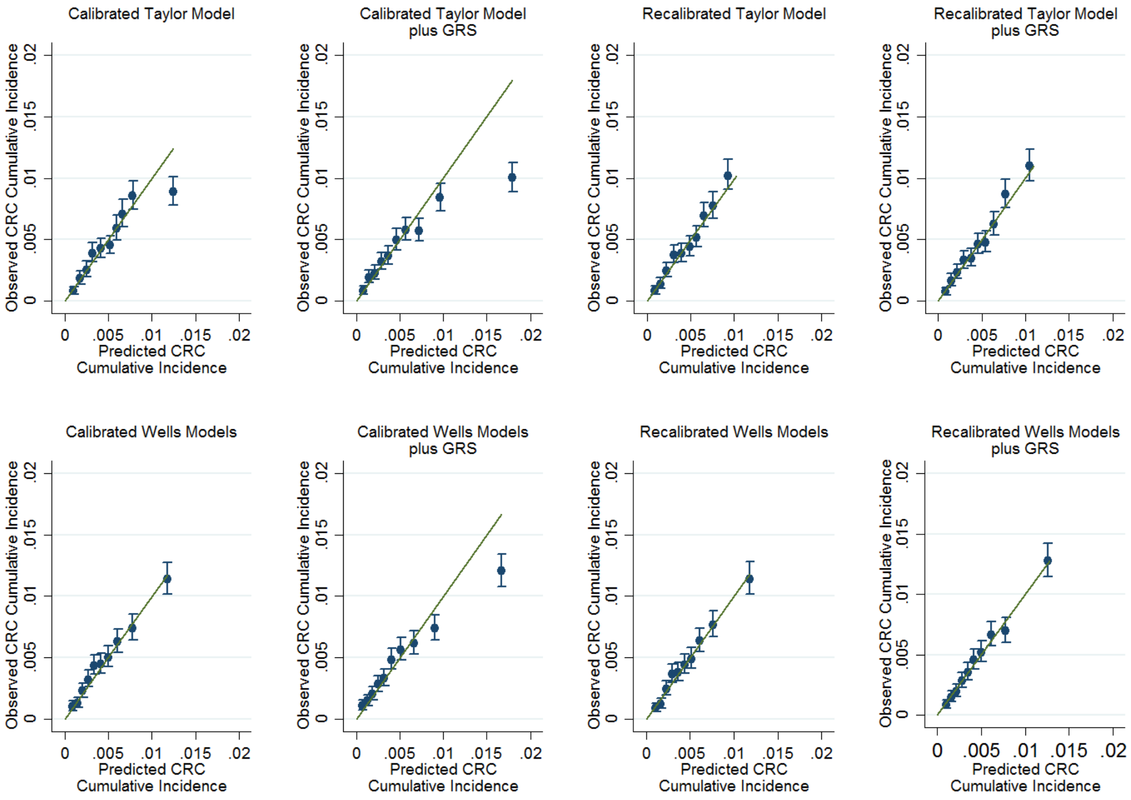

Supplementary Figure 6: Calibration plots for the Taylor et al. [5] and Wells et al. [6] models in the UK Biobank, restricting the analysis to participants available for both the Taylor et al. and Wells et al. models, and excluding 1st and 2nd degree relatives. The original models were initially calibrated to the UK Biobank population and following this the genetic risk score (GRS) was combined with the model's original coefficient(s). To ensure comparable calibration between models with and without the GRS, we then further recalibrated by the predicted log hazard from the original model as a covariate in a flexible parametric survival model by itself, and with the addition of the GRS.

References

1. Smith T, Muller DC, Moons KGM*, et al.* Comparison of prognostic models to predict the occurrence of colorectal cancer in asymptomatic individuals: a systematic literature review and external validation in the EPIC and UK Biobank prospective cohort studies. Gut 2018, http://gut.bmj.com/content/early/2018/04/03/gutjnl-2017-315730.abstract.
2. Sudlow C, Gallacher J, Allen N*, et al.* UK Biobank: An Open Access Resource for Identifying the Causes of a Wide Range of Complex Diseases of Middle and Old Age. PLoS Medicine 2015;12(3):e1001779.
3. UK Biobank. UK Biobank: Protocol for a large-scale prospective epidemiological resource. Protocol No: UKBB-PROT-09-06 (Main Phase). In; 2007.
4. Bycroft C, Freeman C, Petkova D*, et al.* Genome-wide genetic data on ~500,000 UK Biobank participants. bioRxiv 2017, http://biorxiv.org/content/early/2017/07/20/166298.abstract.
5. Taylor DP, Stoddard GJ, Burt RW*, et al.* How well does family history predict who will get colorectal cancer? Implications for cancer screening and counseling. Genet Med 2011;13(5):385-91.
6. Wells BJ, Kattan MW, Cooper GS*, et al.* Colorectal cancer predicted risk online (CRC-PRO) calculator using data from the multi-ethnic cohort study. J Am Board Fam Med 2014;27(1):42-55.
7. International Physical Activity Questionnaire. Guidelines for Data Processing and Analysis of the International Physical Activity Questionnaire (IPAQ) - Short and Long Forms. In; 2005.
8. Peters U, Bien S, Zubair N. Genetic architecture of colorectal cancer. Gut 2015;64(10):1623-1636.
